# Revealing a Pre-neoplastic Renal Tubular Lesion by p-S6 Protein Immunohistochemistry after Rat Exposure to Aristolochic Acid

**DOI:** 10.15586/jkcvhl.2015.38

**Published:** 2015-09-08

**Authors:** Alexandra Gruia, Patrycja Gazinska, Diana Herman, Valentin Ordodi, Calin Tatu, Peter Mantle

**Affiliations:** 1 Pathology Department, County Hospital Timisoara, Timisoara 300736, Romania; 2 Breakthrough Breast Cancer Research Unit, Guy’s Hospital, King’s Health Partners AHSC, King’s College London School of Medicine, London SE1 9RT, UK; 3 Department of Biology, University of Medicine and Pharmacy “Victor Babes”, Timisoara, Romania; 4 Centre for Environmental Policy, Imperial College London, London SW7 2AZ, UK

## Abstract

Aristolochic acid (AA) has, in the last decade, become widely promoted as the cause of the Balkan endemic nephropathy and associated renal or urothelial tumours, although without substantial focal evidence of the quantitative dietary exposure via bread in specific households in hyperendemic villages. Occasional ethnobotanical use of *Aristolochia clematitis* might be a source of AA, and Pliocene lignite contamination of well-water is also a putative health risk factor. The aim of this study was two-fold: to verify if extracts of *A. clematitis* and Pliocene, or AA by itself, could induce the development of renal or urothelial tumours, and to test the utility of the ribosomal protein p-S6 to identify preneoplastic transformation. Rats were given extracts of *A. clematitis* in drinking water or AA I, by gavage. After seven months, renal morphology was studied using conventional haematoxylin and eosin and immunohistochemistry for ribosomal p-S6 protein. Plant extracts (cumulative AA approximately 1.8 g/kg b.w.) were tolerated and caused no gross pathology or renal histopathological change, with only faint diffuse p-S6 protein (except in the papilla) as in controls. Cumulative AA I (150 mg/kg b.w. given over 3 days) was also tolerated for seven months by all recipients, without gross pathology or kidney tumours. However, p-S6 protein over-expression was consistent particularly within the renal papilla. In one case given AA I, intense p-S6 protein staining of a proximal tubule fragment crucially matched the pre-neoplastic histology in an adjacent kidney section. We briefly discuss these findings, which compound uncertainty concerning the cause of the renal or upper urinary tract tumours of the Balkan endemic nephropathy.

## Introduction

As both a nephrotoxic and a carcinogenic environmental toxin, aristolochic acid (AA), as a constituent of plants of the genus Aristolochia, has in recent years been implicated as the major disease determinant for the Balkan Endemic Nephropathy (BEN). BEN was recognised in the 1950s as a distinct and idiopathic entity in certain rural communities of Bulgaria, Romania and Yugoslavia. Krogh (1) first suggested that the mycotoxin ochratoxin A (OTA), to which a nephropathy occurring in the Danish bacon industry had been ascribed in the 1970s, might also cause the slow silent bilateral renal atrophy of the human BEN. The arable weed plant *Aristolochia clematitis*, which is endemic in parts of Eastern Europe was also suggested as a possible cause of BEN nearly half a century ago (2). However, Aristolochia spp. have long formed part of the oriental *materia medica* in herbal formulations (3) and the general toxicity and carcinogenicity of the principal toxic component, AA, was already well established in experimental animals (4, 5). Concurrently, other aetiological factors are being considered, since the natural occurrence of Pliocene lignite deposits in the Balkans fits rather closely with the geographical distribution of BEN hotspots (6), thereby offering rather strong *prima facie* evidence for exposure to leachate from such deposits into the well-waters on which rural communities rely. However, no lignite component has yet been shown experimentally to mimic any of the BEN pathology.

The putative role of AA in tumours of the renal pelvis and urethra gained attention in the 1990s during the local epidemic of nephropathy in Belgian women. This was attributed to formulation error in a Chinese herbal slimming medication involving accidental inclusion of Asian material in which AA was a characteristic toxic alkaloid. Early attempts to find an experimental animal model to mimic the development of renal or urothelial tumours used female rabbits (7). Intraperitoneal delivery of a mixture of AA I and II (0.1 mg/kg b.w.) 5 days a week to 12 animals for up to nearly 2 years caused marked glucosuria and proteinuria and extensive renal tubular atrophy and interstitial fibrosis in a context of reduced feed intake and poor concomitant growth. One rabbit died, but three developed urinary tract tumours (one small *in situ* renal carcinoma, one tubulopapillary adenoma, and one transitional cell carcinoma in mid-ureter together with an extensive peritoneal papillary malignant mesothelioma. Another report on rat tumourigenicity of AA, yielding kidney neoplasms in all experimental individuals six months after sub-acute insult *per os* while not arising from transitional cell epithelia, is that of Cui et al (8). If AA is an etiological agent for renal or urothelial tumours in BEN, identification of early markers of neoplastic transformation may aid in the diagnosis and management of AA-induced renal/urothelial tumours. In this study, we tested the utility of the ribosomal protein p-S6 (phospho S6 ribosomal protein) as a potential marker for AA-induced renal tumours in rats treated with AA. Further, since our Balkan ^1^H NMR metabolomic study differentiated a BEN cohort in Romania from one in Bulgaria (9), and that we are aware of ethnobotanical exposure to AAs, we have included animals additionally given Pliocene lignite infusion and pilot groups given chronic exposure to ethnobotanical extracts of *A. clematitis*.

Focus on expression of ribosomal p-S6 protein in kidney of rats challenged with AA has arisen from our previous experience with cancers in kidneys of rats given chronic tolerable exposure to dietary OTA (10). That study revealed a consistent specificity for the renal tumours attributable to that environmental alkaloid in contrast to other neoplasms, e.g. testis tumours or subcutaneous fibrosarcoma, of other causation. The OTA findings were reminiscent of those in the spontaneous renal tumours of the Eker rat (11). The Eker strain is heterozygous for a dominantly-inherited germline mutation in the Tsc 2 tumour suppressor gene that is recognised as a valid model for human tuberous sclerosis complex (12), with two genes (TSC1 and TSC 2) involved and tumours occurring sometimes in kidney. Activation of the TSC 2 and folliculin genes in mice has been associated with both renal tumour development and mammalian target of rapamycin (mTOR) dysregulation (13). A few rare human cases of familial renal cell carcinoma have been attributed to disruption of the TSC 2 gene (14). More recently, Wilson et al (15) generated Tsc 1^+/-^mice with predisposition to kidney cancer, and strong staining for p-S6 protein in those tumours. These observations form a rationale for novel pilot exploration of p-S6 as a marker for putative AA-induced renal/urothelial tumours or pre-neoplastic transformation.

## Materials and methods

All animal experiments complied with the European Convention for the Protection of Vertebrate Animals used for Experimental and other Scientific Purposes (Strasbourg, France, 1986). The University of Medicine and Pharmacy Timisoara Ethical Committee approved the experimental protocols. Young female Sprague-Dawley rats were caged in a day-night controlled room, properly ventilated, and with *ad libitum* access to food and water. Animals were observed daily and growth was monitored by weight. Within available resources, several biochemical parameters were monitored at intervals to detect any marked changes. For assessment of liver and kidney function, aspartate aminotransferase (AST), alanine aminotransferase (ALT), creatinine and urea were evaluated. Blood was collected from a femoral vein, allowed to clot for 30 min, centrifuged and processed for serum concentration according to Siemens Diagnostics ALT-AST kit, creatinine kit and urea kit, respectively.

### Experiment 1

This experiment tested aqueous extracts of *A. clematitis*, using six rats (commencing 5 weeks old; 82–137 g) for six months. *A. clematitis* plants had been collected from wild habitats (Western and South-western Romania, May-June, 2006) and left to dry in a controlled humidity room. Dried leaves were powdered with pestle and mortar. Two types of extract were prepared. For two rats, two grams of leaves were Soxhlet-extracted in 200 mL of distilled water for 30 min. For two other rats one gram of leaves was suspended in 200 mL of hot (80 °C) distilled water and left to infuse for 30 min, in a manner reproducing the ethnobotanical decoction preparation of *A. clematitis* in some Romanian rural areas. Two more rats served as controls and were given tap water.

*A. clematitis* leaf aqueous extracts were analyzed by an HPLC method to establish AA concentrations. An analytical standard containing a mixture of AA I and II was purchased from Sigma-Aldrich (St. Louis, CO, USA). Acetonitrile (HPLC-grade) and HPLC grade water were also obtained from Sigma-Aldrich. All experiments in this study were performed with an Agilent 1100 HPLC system. An HP 1100 liquid chromatograph system (Agilent Technologies, Santa Clara, CA, USA) consisting of a binary pump, a thermostat-controlled column and UV detector plus on-line degasser was used. Data were analyzed using the HP Chemstation System. The analytical column was a Zorbax SB-C18 (5 µm, 4.6 x 250 mm) (Agilent Technologies). The eluents consisted of HPLC grade water acidified with phosphoric acid 98 % to a pH value of 3 (A) and acetonitrile (B). The initial condition was set at 20 % B with a gradient to 70 % B in 25 min, then a linear gradient to 100 % B in 30 min. The flow rate was 0.5 mL/min. All analyses were monitored at 390 nm. The column temperature was set at 40 °C, and the sample injection volume was 20 µL. The peak of AA I and II in samples was identified by comparing their retention time values and UV spectra with those of the standard. The aqueous extracts obtained by Soxhlet and hot water infusion were analyzed prior to the beginning of the experiment and AA concentrations were determined. To calculate the concentration of AA, a calibration curve was made based on standard AA dilutions from 1 to 40 mg/100 mL. AA standard dilutions were prepared in acetonitrile. On average, 2.96 mg AAI and II/100 mL was found in the Soxhlet aqueous extract of *A. clematitis* leaves, while the hot water infusion contained on average 2.5 mg/100 mL. Predicted intake volume was 150 ml/kg/day (16), translating into approximately 900 and 750 µg AAs/day, respectively.

The aqueous extracts obtained were filtered through 0.45 µm membrane filters prior to use as drinking water. Extracts were freshly prepared, based on demand, twice a week. Six months later all rats were euthanized for general autopsy, but particularly studying urinary tracts. Kidneys and a liver sample were collected and preserved in 4 % formalin solution for histology. Tissues were embedded in wax blocks and sections (3 µm) stained with H & E by the standard hospital pathology protocol for reviewing in Romania.

### Experiment 2

Six female Sprague-Dawley rats (5 weeks old, 100–125 g) were given AAI (purity >97%; Sigma-Aldrich Corporation, St. Louis, MO, USA) by gavage at a dosage of 50 mg/ kg b.w. Approximately 5.5 mg AAI sodium salt dissolved in 20% ethanol in phosphate buffered saline was administered (1 ml) to each rat on three consecutive days. Thereafter, animals were maintained on standard rat diet for a further seven months at an average body weight of 255 g.

Three female Sprague-Dawley rats weighing 150–170 g were administered AA I in solution in a Pliocene lignite aqueous extract. This extract was prepared from coal samples collected from the Husnicioara open pit mine (South-western Romania) in September 2011. A Soxhlet method was used for extraction, with 10 g of coal and 250 mL bi-distilled water for 5 days; the aqueous extract had a brownish colour and was used as a solvent for dissolving the AAI (purity >97%, Sigma-Aldrich, St. Louis, MO, USA). The gavage solution (1 mL) delivered approximately 5.5 mg AAI sodium salt/rat/day on three consecutive days. Thereafter, animals were maintained in standard (control) conditions for six months. Resource constraints focused all available animals in experiment 2 on treated groups; general controls were those in experiment 1, noting that in (8) 22 control rats had been without disease throughout.

### Immunohistochemistry for p-S6 protein

The protocol was as previously described (10) and included fresh H & E – stained adjacent sections for direct comparison with those stained immunohistochemically. In brief, immunostaining of 3 µm sections was with Vectastain Elite ABC kit (PK-6101). In addition, avidin/biotin block (Vectastain SP-2001) was applied prior to the primary antibody (polyclonal phospho-S6 protein [Ser240/244, Cell Signalling #2215] in 1:200 dilution). Sections were developed using DAB, counterstained in Gill III haematoxylin, dehydrated and mounted with DPX. Immunostained, and standard haematoxylin and eosin (H & E)-stained sections were scanned (Hamamatsu Nanozoomer) and stored on the digital slide server (DSS) in ndpi format for reviewing using Digital Images Hub (Slidepath system) for online validation and record.

## Results

### Experiment 1

**Figure 1.**
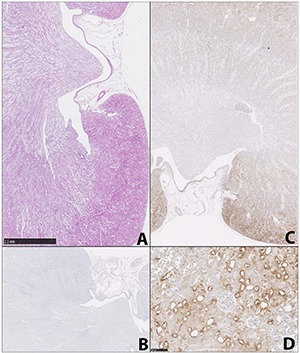
Control (untreated) kidneys; **A**, haematoxylin and eosin staining showing normal histology (bar 2.5 mm); **B**, Negative immunohistochemical test for p-S6 in untreated kidney; **C**, diffuse weak staining for p-S6 in cortex but none in papilla; **D**, tangential section through renal cortex typical of treated rats illustrating diffuse staining for p-S6 (applicable also to untreated controls as in C), D (bar 100 µm).

None of the rats showed any adverse reaction to the treatments, no abnormalities were evident at necropsy, and no H & E histopathology was evident in longitudinal sections of kidney (as also for those stained in London; **Figure 1A**), or in liver (not shown). Concerning use for the first time of an immunohistochemical probe in AA toxicology, a negative control for p-S6 protein antibodies (secondary antibody only) showed no staining (**Figure 1B**). Consistent features in all control and treated rats were that in all kidneys sectioned through the papilla that region was not stained (**Figure 1C**), as in the negative control. There was no evidence of proliferation of the transitional cell layer in the renal pelvis and there was also no staining for p-S6 protein there. However, controls had variable diffuse staining from inner medulla to cortex that seemed to be confined to vascular elements but this did not involve glomeruli (**Figure 1D**); the reason for this distinction is unclear, but in BEN renal atrophy glomeruli are generally well preserved. In the cortex of the group receiving Soxhlet extract of leaves, diffuse staining of p-S6 was observed. The group receiving hot water extract also showed patchy diffuse staining for p-S6 protein in cortex and medulla, but there was no strong basis for perceiving significant differences attributable to either treatment. Overall, there was no macroscopic or microscopic evidence of tumour growth in response to plant extracts, and the diffuse staining pattern of p-S6 was not markedly different between groups.

### Experiment 2

Rats given AA I alone were 100–125 g (mean 120 ± 6.4 g). After one month the mean was 186 g, reaching 255 g at seven months, all conforming to standard growth data (17) and showing no persistent reaction to the treatment. Blood plasma creatinine concentration increased from the puberty value at the start (0.33 ± 0.06 mg/dL) to 0.45 ± 0.05 mg/dL a month later and was sustained at 7 months (0.44 ± 0.06 mg/dL). Plasma urea was 30 ± 11 mg/dL at the start, 28 ± 10 mg/dL after one month and 33 ± 10 mg/dL at 7 months, not significantly different. Both hepatic enzymes declined slightly in concentration over the period, but values remained within normal range. Animals also given Pliocene lignite infusion as gavage were initially 150–170 g (mean 164 g). At term, six months later, their mean weight was 240 g, similarly typical for this breed. Over the treatment period plasma creatinine (0.45 ± 0.02 and 0.47 ± 0.06 mg/dL) and urea (32 ± 8 and 25 ± 3 mg/dL) values were maintained, AST was unchanged and ALT decreased only slightly, though within the normal rat range. As in experiment 1, no significant histopathological change was evident in initial H & E sections. In both experiments, ureters and bladders were viewed at necropsy for gross change; none was found and so tissues were not embedded for histology.

**Figure 2.**
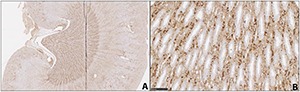
p-S6 staining in AA treated kidneys. **A**, AA I treated kidneys showing staining for p-S6 protein principally in the papilla, contrasting with Experiment 1; **B**, detail in papilla (bar 100 µm).

**Figure 3.**
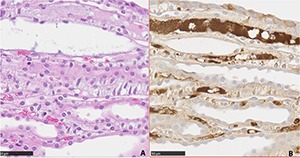
Closely matching longitudinal sections through renal papilla stained with haematoxylin and eosin (**A**) or for p-S6 protein (**B**) correlating stained areas, probably vascular (note erythrocytes), between some ducts and tubules. Bar 50 µm.

Across the two parts of the semi-acute AA I experiment, immunological distribution of p-S6 protein in kidneys contrasted with that in the chronic plant extract experiment 1. There was extensive interstitial staining in the papilla (**Figure 2 A, B**), sometimes more clearly defined where the preparation was slightly oblique to the central sagittal plane, showing tubules more in transverse section. However, elsewhere staining could be matched in H & E preparation (**Figure 3A**) with that of p-S6 to amorphous matrix between tubules (**Figure 3 B**). Notably also, diffuse weak staining occurred in cortex, usually emphasising the glomeruli, and contrasting with absence in the medulla (as in **Figure 1**). There was consistent absence of cellular and nuclear proliferation in the transitional cell layer lining the renal pelvis, and of staining for p-S6 protein. Distribution of apparent p-S6 protein over-expression in response to AA I did not differ with inclusion of Pliocene lignite infusion in the gavage dosing vehicle.

**Figure 4.**
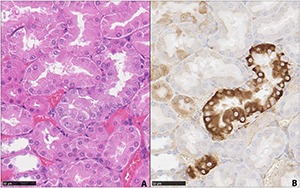
Seven months after oral gavage with AA. Adjacent sections in renal outer stripe of the outer medulla stained with haematoxylin and eosin (**A**), or immunohistochemically for expression of p-S6 protein (**B**). Stained nephron fragment(s) are interpreted as indicative of a pre-neoplastic lesion (bar 50 µm).

Immunohistochemistry was crucial for recognising a few specific features attributed to renal tumourigenesis. Paucity of p-S6 protein staining in the outer stripe of the outer medulla (OSOM) of one rat in the AA I only treatment group, in which papilla staining is illustrated in Figures 2 and 3, enabled observation of a small proximal tubule element with intense staining for p-S6 protein (**Figure 4**); positional matching to an adjacent section stained by H&E revealed a focus of prolifer-ation (**Figure 4A**). That staining revealed the epithelium of this proximal (straight segment) tubule containing a higher concentration of cells than in adjacent tubules; the cells were crowded, appearing hyperplasic or proliferating. A few nuclei were slightly larger than others (karyomegaly) and all of them contain nucleoli. Compared to the other tubules in the region, which had nuclei spaced far apart, there was a tendency in the particular tubule fragment to lose polarity, to initiate a sort of stratification. Less prominent, the cytoplasm in the cells that had enlarged nuclei seemed to have lost some eosinophilia to become clearer. Brush borders also seemed to be deficient. Ribosomal p-S6 protein staining in the tubule centre is intense and diffuse within the cytoplasm, as was also the edge of another nearby tubule fragment, possibly of the same tubule (**Figure 4B**). Other proximal tubules in the region either had no staining or showed just a weak, apically-concentrated fine granular reaction.

## Discussion

In spite of six months of exposure to AAs in drinking water there was no detectable adverse clinical or histopathological effect. The mild, diffuse over-expression of ribosomal p-S6 protein in renal cortex of controls seems to be at least partly an inconsequential feature of the local water supply or of commercial rat diet, which had not been evident in previous UK studies in London (10). However, the *A. clematitis* infusions, prepared in distilled water, did not significantly affect the intensity of immunohistochemical staining. Notably, the cumulative intake of AA (~1.8 g/kg b.w.) was three-fold higher than that given during half the exposure period (18) and which had caused extensive histopathological changes. Therefore, the present pilot study suggests that, for environmental toxins, it is futile to rely on convenient once-daily gavage or parenteral injection for other than pharmacokinetic studies, because environmental AA seems to be rather well-tolerated in rats if given slowly throughout each day. Humans may be similar. In contemplating what might be necessary for *A. clematitis* to cause BEN and its tumours, the amount may exceed any realistic human exposure.

The semi-acute high-insult administration of AA I was adopted partly to mimic the end-point outcome as described previously (8). Cui et al. (8) had used AA I (> 95 % purity) isolated from *Aristolochia manshuriensis* Kom, presumably not different from the commercial AA I used here although there is recent concern (19) that highly mutagenic AA analogues remain to be recognised in some species. Of the 14 rats used, 4 developed unilateral kidney tumours, and the remaining animals showed pre-neoplastic renal lesions. Lesions were small nodules, the smallest of which were already 2–3 mm in diameter, while larger ones sometimes extended through the renal capsule. We were unable to reproduce these results, despite prolonging the experiments for seven months instead of six. Clearly, our present concept of a pre-neoplastic lesion is very different (see below).

Interesting comparison can be found in the study of Schmeiser et al. (18) which used Wistar males, gavaged daily at 10 mg AA I/kg b.w., sodium salt in water for 3 months. The experiment terminated 7 months after starting, as in our present experiment. Among 18 treated rats, most gave squamous cell carcinomas in the forestomach and sometimes also in an ear duct. Other tumours occurred in the small intestine and /or the pancreas. One occurred in a kidney (not studied). Although the treatment had been widely carcinogenic there were no transitional cell tumours. Similarly, even Ivic (2) who first perceived a role for AA in the kidney atrophy of BEN, did not find experimental urothelial tumours. The cumulative dose in (18) had been at least that in experiment 2 here, although gavaged 5 days/week over a much longer period. Clearly there is need for experiments on short and long-term exposure to AA at a range of moderate doses, preferably given in feed (20) and based on US National Toxicology Programme-like rigour (21), to establish the plausible risk of renal cancer particularly in transitional cells of the kidney pelvis.

Although we did not observe renal tumours or lesions, we observed features of pre-neoplastic transition in response to AA in some areas of the kidney. There was a close match between p-S6 staining and morphological change as observed by H & E staining. The principal value of immunohistochemical staining for p-S6 protein hyper-expression has been the highlighting, for the first time, of a small proliferation in the epithelium of proximal straight tubule segments of nephron located in the OSOM, revealing and matching what had not been recognised otherwise in a closely-adjacent conventionally stained section. This histopathological change represents our concept of a pre-neoplastic proliferation and is reminiscent of the periphery of a matching small neoplastic lesion in a rat given protracted exposure to dietary OTA (Figure 2A) in 10). Our concept is also consistent with a literature illustration (22). This lesion did not appear to be resolving seven months after the AA insult and thus could have been a sufficient focus for subsequent proliferation towards kidney cancer.

Extensive serial sectioning and immunohistochemical staining might have been expected to reveal other examples, if resources had been available. The utility of p-S6 as a marker of pre-neoplastic renal tubular lesions warrants further investigation, but the important feature of the present findings is that of AA-initiated potential carcinoma arising in renal parenchyma, as is the case with another nephrotoxin OTA, but not by proliferation in the transitional cell epithelium of the renal pelvis. To demonstrate the latter experimentally as evidence for AA causing the urological tumours in BEN patients is epidemiologically-desirable to satisfy classical (23) and modern (24, 25) criteria. Currently, human AA exposure evidence for BEN is conflicted (15, 26, 27) and claims that AA is the disease determinant (28–30) appear premature.
